# The raising threat of CCHF in Afghanistan: Healthcare dilemmas and the need for comprehensive reponses

**DOI:** 10.1016/j.nmni.2023.101198

**Published:** 2023-11-21

**Authors:** Ahmad Neyazi, Moeed-ul-Haq Fakhri, Nosaibah Razaqi, Habibah Afzali, Prakasini Satapathy, Sarvesh Rustagi, Bijaya Kumar Padhi, Mozhgan Ahamdi

**Affiliations:** Afghanistan Center for Epidemiological Studies, Herat, Afghanistan; Infectious Disease Ward, Herat Regional Hospital, Herat, Afghanistan; Afghanistan Center for Epidemiological Studies, Herat, Afghanistan; Center for Global Health Research, Saveetha Medical College and Hospital, Saveetha Institute of Medical and Technical Sciences, Saveetha University, Chennai, India; School of Pharmacy, Graphic Era Hill University, Dehradun 24800, India; School of Applied and Life Sciences, Uttaranchal University, Dehradun, Uttarakhand, India; Department of Community Medicine and School of Public Health, Postgraduate Institute of Medical Education and Research (PGIMER), Chandigarh, India; Afghanistan Medical Students Association, Herat, Afghanistan

Dear editor

Afghanistan is currently facing, exacerbated by the alarming surge in Crimean-Congo hemorrhagic fever (CCHF) cases [1]. This week, the Centers for Disease Control and Prevention (CDC) issued a Level 1 travel notice, advising individuals to practice usual precautions when traveling to Afghanistan due to the widespread CCHF outbreak across most provinces [2]. From the onset of 2023 until September 20, 2023, a total of 977 individuals exhibiting symptoms indicative of infection were subjected to Polymerase Chain Reaction (PCR) testing, which confirmed 352 cases [1]. The positivity rate was computed at 36.0 ​%, and the mortality rate was observed at 9.7 ​% [1]. In the preceding four months, a notable surge in the CCHF-associated mortality was observed, with Kabul and Balkh provinces bearing the brunt, reporting 48 and 15 cases respectively during this timeframe [1,3]. Nonetheless, the contagion has permeated other regions, including Takhar province, engendering apprehensions regarding the dire repercussions and the challenges encountered in healthcare provision. This paper elucidates the Crimean-Congo Hemorrhagic Fever (CCHF) outbreak in Afghanistan, delves into the virus's distinctive attributes, examines the broader healthcare dilemmas confronting the nation, and underscores the pressing exigency for comprehensive healthcare interventions [3] (see Fig. 1).

**Fig. 1:**
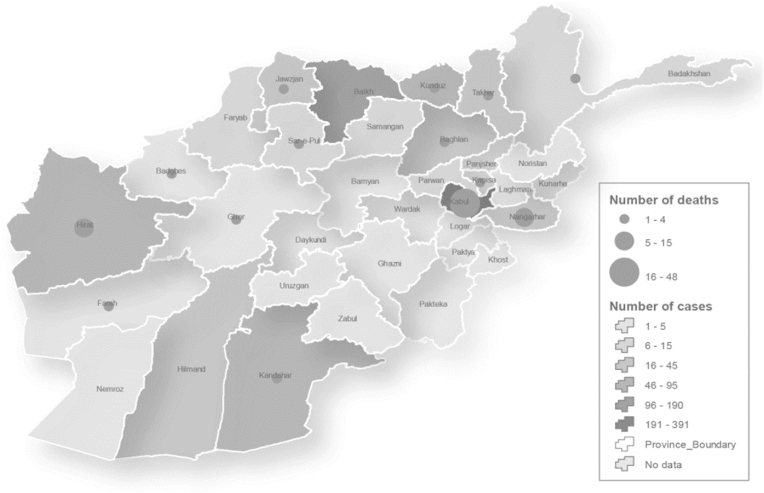
Geographical Distribution of Crimean-Congo Hemorrhagic Fever (CCHF) cases by Province in Afghanistan, 01 Jan–16 Sep 2023.

Crimean-Congo Hemorrhagic Fever (CCHF), attributable to the Crimean-Congo Hemorrhagic Fever virus (CCHFV), is a tick-transmitted pathogenic agent classified within the Nairoviridae family in the Bunyavirales order [4]. The virus was initially characterized in the 1940s, following occurrences of hemorrhagic illness among Soviet military personnel stationed in Crimea [4]. Subsequently, during the 1960s, a virus exhibiting analogous clinical presentations was identified in the Belgian Congo, prompting the nomenclature CCHFV [4]. This virus possesses a genome consisting of three single-stranded RNA segments and is recognized to be in circulation in diverse regions across Africa, Asia, and Europe, especially in locales where Hyalomma spp. ticks are prevalent [5].

The transmission of CCHFV entails both wild and domestic animals serving as hosts for ticks [5]. These animals serve as sources of blood meals for ticks and can facilitate the transmission of the virus to both ticks and humans during viremic phases. Investigations into seroprevalence have identified CCHFV-specific antibodies in a range of animals, including livestock, horses, dogs, chickens, camels, among others [5]. Human acquisition of CCHF can occur through tick bites, incidents of needle-stick injuries, or direct contact with infected blood. The majority of CCHF cases are either devoid of symptoms or present with mild clinical manifestations, whereas severe cases exhibit hemorrhagic features and may experience heightened vascular permeability and an elevated cytokine response [5]. The illness is characterized by symptoms akin to those of influenza, and in severe instances, patients may manifest petechiae, epistaxis, and profuse bleeding from various bodily systems [5].

Afghanistan grapples with a myriad of health challenges, including the evacuation of over 124,000 civilians, including healthcare providers, since mid-2021, significantly impeding the delivery of healthcare services [5]. Poverty, food insecurity, ongoing conflict, severe drought, and the lingering effects of the COVID-19 pandemic have exacerbated the situation, disproportionately affecting vulnerable groups, notably women and children [5].

The recent Taliban-imposed ban on girls' education has led to a shortage of female health workers, further restricting access to critical healthcare services for women and children. This constraint exacerbates the impact of diseases like CCHF, placing comprehensive healthcare delivery in jeopardy [5].

The surge in CCHF cases is a culmination of disruptions in healthcare services, socioeconomic challenges, and population movement, coupled with transmission from animals to humans. To effectively combat this outbreak, a comprehensive approach is vital, encompassing heightened surveillance, education, and public health measures. Ensuring an ample healthcare workforce, including female health workers, is essential to meet the specific needs of vulnerable populations.

International support and collaborative efforts are imperative to alleviate the double burden faced by Afghans under Taliban rule. The CDC's travel advisory underscores the gravity of the situation and highlights the urgency for immediate action.

The surge in CCHF cases in Afghanistan is an unprecedented challenge, particularly for women, children, and other vulnerable groups. Urgent measures are essential to address the outbreak, fortify healthcare delivery, and provide crucial services to those in need.

## Author contribution

All authors are equally contributed.

## Sources of funding

None.

## Ethical approval

N/A.

## Consent

N/A.

## Declaration of competing interest

The authors declare no conflict of interest.
